# A new immunology forum for a new age of immunology

**DOI:** 10.1093/oxfimm/iqaa002

**Published:** 2020-06-18

**Authors:** Daniel M Altmann

**Affiliations:** Imperial College, London

These are unprecedented times in many ways. The COVID-19 pandemic and lockdowns have been a time of challenge and evolution for everyone, not least immunology and immunologists. Most explicitly, there has been the overwhelming clinical and scientific imperative for all to step up to the plate and put our skillset to use in the international efforts to address the infection. Along with the urgent and insatiable appetite for data in real time has come a need to rethink the terms of engagement in scientific publishing: the need to be timely, responsive and accessible and yet retain the highest academic standards and rigour. As the newly appointed Editor-in-Chief among the team at *Oxford Open Immunology*, the newest immunology journal, we have given much thought to what an immunology journal needs to look like to meet the specific needs of our community in 2020 and into the future.

The launch of a new immunology journal for the Oxford University Press (OUP) collection is an opportunity to start with a *tabula rasa* and build a relevant, new, journal for new times. For any unfamiliar with OUP, it is a publishing brand with an impeccable, unmatched heritage in academic publishing—its published history runs to three volumes and 2250 pages. OUP is the second oldest academic publisher and the largest University Press in the world. OUP now publishes more than 450 academic journals, with a publishing presence in more than 100 countries.

So, building on that incredible tradition, the challenge now is to deliver a vibrant new journal that can satisfy unmet needs for the research community. What could these unmet needs be if there are already greater than 150 immunology journals in existence? Many of the current benchmarks indeed cut across several immunology journals: we all now look for a journal that will be fully open access and compliant with DORA (San Francisco Declaration on Research Assessment) guidelines, which will be truly accessible, rapidly responsive, fair and genuinely international without attachment to any individual immunology research community or to native-English speakers.

With the international scope in mind, we have a core team of associate editors based in the UK, USA and China, and are assembling an outstanding, multi-disciplinary editorial board, covering immunology across some 14 countries. Most all, we are keen that the passions and skillset of those at the journal must be in tune with the emergent questions and studies out there in the community. All of the traditional, core topics of cellular subsets, functions, regulation, antibody, cytokine and complement characterization, disease studies and infection will need to be accompanied by developing areas and newer technologies including single cell and structural biology approaches, informatics and artificial intelligence, imaging and mass cytometry. If you have ideas for areas you would like to see included, I urge you to get in touch.

Having spent greater than 20 years as an editor at various immunology journals, I have observed the changing trends in immunology publishing and have also had the good fortune to make many lasting friends in the course of commissioning review articles. Outstanding review articles will here form an important part of our content. We are all ever busier and groan when invitations pop up in the inbox asking for yet another review article, so what are they for and why say ‘yes?’ Think of your favourite review articles in your field and the answer will be clear. A great review article is far more than a me-too repackaging of recently published papers. At best, these pieces are where we do our real thinking, with the expanse to apply some genuine brain-wattage to synthesis of what has been happening, how it fits together and where we should be heading. We welcome submissions from all who are excited at the thought of rising to this challenge.

With the space and immediacy of our online identity, we are well-placed to respond to these fast-moving times for immunology and the associated technologies. For my taste, some of the most vibrant areas are the online chatrooms where new strategies are thrashed out, whether for high-dimensional flow analysis, NGS informatics or microbiota pathway analysis. Our aim is to instil a sense of working with the international research community by offering a single home in which to find key protocols, methodologies, debate and discussion. So, much of immunology publishing now comprises the *magnum opus*, resulting from the combined efforts of dozens of people working for several years. While that is important and laudable, we also want to be able to offer a home to the first publications of the enthusiastic grad student or post-doc who has written a really useful piece of code to solve a problem in data set handling, or who has a new trick for immunophenotyping in small samples or multiplexing peptide panels.

The least we can do is offer to supply a scientist-led, welcoming experience where we will aim to deal efficiently, fairly and honestly with your precious data and manuscripts. Please send them in. As many generations of students have heard in my lab meetings, a scientist who does experiments and then leaves them festering, unpublished in their lab notebook is no different to a baker who gets up to bake bread but then does not get around to feeding anyone with it. Get in touch!

